# *In Vitro* Antioxidant and* In Vivo* Lipid-Lowering Properties of* Zingiber officinale* Crude Aqueous Extract and Methanolic Fraction: A Follow-Up Study

**DOI:** 10.1155/2019/9734390

**Published:** 2019-07-09

**Authors:** Oussama Bekkouch, Mohamed Harnafi, Ilham Touiss, Saloua Khatib, Hicham Harnafi, Chakib Alem, Souliman Amrani

**Affiliations:** ^1^Laboratory of Biochemistry and Biotechnologies, Department of Biology, Faculty of Sciences, 60050 Oujda, Morocco; ^2^Biology Department, Sciences and Technologies Faculty, Moulay Ismail University, 52000 Errachidia, Morocco

## Abstract

Over the past decades, cardiovascular diseases have become the leading cause of death all over the world, and among these diseases there is atherosclerosis caused mainly by an increase in plasmatic cholesterol levels and by strong oxidation caused by free radicals. For these reasons and others, we explored in this report the hypolipidemic and the antioxidant effects of* Zingiber officinale* crude aqueous and methanolic extract. The hypolipidemic study was carried out in high-fat-fed mice model. Animals were subdivided into four groups and were orally treated with the aqueous extract once daily for twelve weeks at two doses: 250 and 500 mg/Kg. During the treatment, the body weight, total cholesterol, triglycerides, and high-density lipoproteins have been defined every four weeks. The antioxidant activity has been studied using radical scavenging activity, *β*-carotene bleaching, reducing power assay, and the TBARs tests. The daily oral administration of the extracts for twelve weeks significantly improved the lipid profile in a dose-dependent manner, from the first until the twelfth week, and also showed a significant antioxidant effect. These findings may be potentially contributive to the validation of the medicinal use of* Z. officinale* to treat hyperlipidemia and cardiovascular complications.

## 1. Introduction

Cardiac pathologies are frequently the first death cause in the world. According to the World Health Organization, 31% of global deaths in 2012 were consequent of cardiovascular affections (World Health Organization, 2015). In the 21st century, lifestyle and food quality have changed, the sedentary lifestyle is more frequent, and high fat/high sugar foods more consumed by the global population, which make them exposed to hyperlipidemia [1]. Hyperlipidemia is a potent risk factor for cardiovascular diseases [2]. Hypercholesterolemia and increased lipoproteins blood level, especially low-density lipoproteins cholesterol (LDL-C), are directly involved in atherosclerogenesis and subsequently* atheroma* plaque genesis [3]. LDL-cholesterol is a highly oxidizable compound. Redox imbalance events promote the increase of oxidized LDL-C (ox-LDL) which is involved in the pathogenesis of several diseases, among which are the cardiovascular diseases like dementia and diabetes mellitus (Aytaç et al., 2008), which significantly contributes in atherosclerogenesis [4, 5]. Targeting hyperlipidemia is an essential therapeutic path to protect or attenuate the development of atherosclerogenesis [3]. For example, Atorvastatin (antidyslipidemic drug) acts by reducing plasmatic lipid levels [6, 7].

Unfortunately, the drugs used to treat or prevent atherosclerosis may have serious adverse effects. Moreover, the development of additional treatments for controlling lipid levels remains necessary to reduce cardiovascular diseases in parallel to conventional medicaments; that is why phytotherapy could be a suitable alternative or a complement to traditional therapy used for the treatment of hyperlipidemia and cardiovascular diseases worldwide [8]. In Morocco, like a lot of developing countries, most patients suffering from hyperlipidemia utilize traditional pharmacopeia to deal with their health problems. In this regard, many medicinal plants have become very important and have shown potent plasma lipid levels-lowering activities [9].

*Z. officinale Roscoe* (Zingiberaceae) is a cosmopolitan plant used around the world for many purposes. For centuries, ginger roots had been used as a spice and as an essential ingredient in medicinal preparations to treat various physiological disorders like rheumatism, nervous diseases, asthma, stroke, and diabetes [10–12]. Latterly, it has been reported that ginger roots extracts express anti-inflammatory [13], antioncogenic [14], and antiemetic effects [15] and antihypercholesterolemic effect as well [16].

Nevertheless, the use of ginger roots in Morocco is limited to culinary activities. Therefore, we aim, in the present study, to assess the* in vitro* antioxidant potentials of ginger root extracts and their effect on plasmatic, hepatic, and fecal lipid profiles* in vivo* in the high-fat diet induced hyperlipidemia in mice for 12 weeks, with follow-up every four weeks.

## 2. Materials and Methods

### 2.1. Plant Material

Rhizomes of* Z. officinale *were purchased from an herbalist in Oujda city. The taxonomic identification of the plant was performed by Professor Fennane Mohammed, a botanist from the scientific institute of Rabat, Morocco. A voucher specimen was deposited in the Herbarium of Faculty of Sciences, University Mohamed First (Oujda, Morocco), under the reference number (HUMPOM-352).

### 2.2. Preparation of Plant Extracts

*(a) Crude Aqueous Extract*. The ginger roots were cut into tiny pieces then mixed in a blender; the obtained powder was infused in bidistilled water for 30 min. After being filtered, the resulting solution was concentrated using a rotary evaporator under vacuum at 60°C. The obtained crude extract was dried and stored at −20°C until use.

*(b) Methanolic Fraction*. After being cut into small pieces, the ginger roots were defatted in Hexane, using a Soxhlet apparatus, then the residual plant material was air-dried and transferred to Dichloromethane (polarity index P_*0 *_= 3,1), which undergo a massive extraction (12 h), for obtaining a liposoluble extract. The remaining plant material was air-dried once more and extracted (12 h) with Ethyl Acetate (P_0_ = 4,4) until getting a somewhat soluble extract. After that, the residual plant was further removed with methanol to obtain the methanolic fraction (P_0_ = 5,1) and then stored at −20°C until use.

### 2.3. Chemicals

The following reagents were purchased from Sigma Chemical Co. (Taufkirchen, Germany): Folin-Ciocalteu, Gallic Acid, Ascorbic Acid, Quercetin, DPPH (1,1-diphenyl-2- picrylhydrazyl), Sodium Hydroxide (NaOH), Sodium Nitrate (NaNO_3_), Aluminum Chloride (AlCl_3_), *β*-carotene, Butylated Hydroxyanisole (BHA), Linoleic Acid, Tween-80, Potassium Ferricyanide [K_3_Fe(CN)_6_], Trichloroacetic Acid (TCA), n-Butanol, Methanol, Ethanol, Chloroform, Cholesterol, Deoxycholic Acid, Sodium Phosphate (Na_3_PO4), Sodium Phosphate dibasic (Na_2_HPO_4_), Sodium Phosphate monobasic (NaH_2_PO_4_), Sodium Carbonate (Na_2_CO_3_), Ferric Chloride (FeCl_3_), Triton WR-1339, Thiobarbituric Acid (TBA), and Copper Sulfate (CuSO_4_).

### 2.4. Determination of Total Phenolic Content

The total polyphenols contents of* Z. officinale* extracts were defined according to the Folin-Ciocalteu colorimetric method [17]. An amount of 1 mL of Folin-Ciocalteu reagent (0.2 N) was mixed with 0.2 mL of each extract. After 5 minutes incubation at room temperature, 0.8 mL of aqueous sodium carbonate solution (7.5%) was added to the mixture. All samples were thoroughly stirred, then the absorbances were recorded after 1 hour, at 760 nm, against a blank containing 0.2 mL of methanol, 1 mL of Folin-Ciocalteu reagent, and 0.8 mL of an aqueous solution of sodium carbonate (7.5%). Gallic acid was used to generate a calibration curve, which allowed the estimation of total polyphenols quantity, as gallic acid equivalents. The total polyphenol content was expressed as an mg gallic acid/g plant extract. All measures were carried out in triplicate.

### 2.5. Determination of Flavonoid Contents

The flavonoid content spectrophotometrically was defined by using a procedure that depends on the formation of a flavonoid-aluminum complex, which has an absorption maximum at 430 nm. The assay was performed according to Chen et al. [18] with some changes. An amount of 1 mL of bidistilled water and 50 *μ*l of Sodium Nitrate (NaNO_3_, 5%* w/v*) was added to 0.2 mL of each extract (0.5 mg/mL). After 6 minutes, 120 *μ*L of Aluminum Chloride (AlCl_3_, 10%* w/v*) was added to the mixture. After 5 minutes, 400 *μ*L of NaOH (1 M) was then admixed to the mix. The absorbances were registered at 430 nm, against a blank consisting of 0.2 mL of bidistilled water, 50 *μ*L of NaNO_3_ (5%), and 120 *μ*L of AlCl_3_ (10%). Quercetin was used as the standard to obtain the calibration curve, and the flavonoid content was determined as quercetin equivalents and expressed as mg quercetin /g of plant extract. As soon as the emulsion has been added, all samples were explored in the spectrophotometer at 470 nm (t_0_), and then BHA has been used as the standard antioxidant compound. All determinations have been carried out in triplicate.

## 3. Antioxidant Assay of Ginger Roots Crude Aqueous and Methanolic Extract

### 3.1. DPPH Free Radical Scavenging Activity

The antioxidant potentialities, mainly radical scavenging activities, were evaluated at the same time as the basic and essential test, due to the harmful effect of free radicals in foods and human tissues [19].

DPPH (1,1-diphenyl-2- picrylhydrazyl) is a well-known radical and represents an efficient “scavenger” for other radicals. It represents a rapid and reliable tool for the estimation of the antioxidant ability of the components of the extract through their ability to deliver H-atoms and electrons [20]. The DPPH assay determined the free radical scavenging ability of the samples, according to De La Rosa, Alvarez-Parrilla, and Shahidi [21] with some modifications. Three concentrations were prepared for each example. An amount of 0.5 mL of each sample solution was mixed with 1 ml of a newly made methanol solution of DPPH (4 mg/100 ml). The samples were incubated for 30 minutes in darkness and at ambient temperature; then, the absorbance was measured with a spectrophotometer at 517 nm. Ascorbic acid was used as a standard. All measures were performed in triplicate.

(1) Radical  scavenging  activity%=A0−A−AbA0×100

where *A*_0_ represents the absorbance of DPPH solution without sample; *A* represents the absorbance of the test sample mixed with DPPH solution, and *A*_*b*_ represents the absorbance of the sample without DPPH solution.

### 3.2. *β*-Carotene Bleaching Test

The concept of this technique is based on the loss of the characteristic orange color of the hydrophobic linoleate /*β*-carotene emulsion, which is mainly induced by free radicals. The free radicals production was triggered by the action of natural oxidation of fatty acids [22] and by thermic inductance, typically at 45°C. A solution of *β*-carotene was prepared by dissolving a quantity of 2 mg in 10 mL of chloroform. After that, 20 mg of linoleic acid and 200 mg of the emulsifier Tween-80 were admixed with the *β*-carotene solution. After removing the chloroform at 40°C from the final solution, 100 ml of distilled water was added to the flask with vigorous stirring. 0.2 mL of this emulsion has transferred into different test tubes containing the sample solution. The tubes were incubated in a water bath at 50°C for 2 hours with continuous shaking. Immediately after the addition of the emulsion, the first absorbance of samples was recorded (t_0_) and then after 2 hours, both at 470 nm. BHA was utilized as a standard. All measures were performed in triplicate.

The inhibition of the lineolate/*β*-carotene radical was calculated using the following formula:

(2) Bleaching  inhibition%=100−initiaialβ−carotenet0−β−caroteneafter  2 hinitiaialβ−carotenet0×100

### 3.3. Determination of Ferric Reducing Power Assay

The ferric reducing activity of our extracts was perdormed according to the method described by Dehpour, Ebrahimzadeh, Seyed Fazel, and Seyed Mohammad [23], based on the reduction of Fe^3+^ present in the K_3_Fe(CN)_6_ complex in Fe^2+^. Different concentrations of the extracts were prepared. A quantity of 0.5 ml of each sample extract was mixed with 1.25 mL of phosphate buffer (0.2 M, pH 6.6) and 1.25 mL of potassium ferricyanide [K_3_Fe(CN)_6_] (1%* w/v*). The mixture was incubated at 50°C for 20 min. After cooling at ambient temperature, the reaction stopped by adding 1.25 mL of Trichloroacetic acid (10%* w/v*). Then the blend was centrifuged at 3000 rpm for 10 minutes. An aliquot of 1.25 mL of the supernatant solution was mixed with 1.25 mL of bidistilled water and 0.25 mL of a solution of ferric chloride (0.1%* w/v*). The absorbance was measured at 700 nm against a blank containing bidistilled water instead of the extract solution. Ascorbic acid was used as a reference compound such that the absorbance was quantified under the same conditions as those of the extract. All measures were performed in triplicate.

### 3.4. Evaluation of Lipid Peroxidation by Assaying Thiobarbituric Acid Reactive Substances (TBARS): Dosage of Malondialdehyde

The malondialdehyde (MDA) represents an oxidative decomposition product of unsaturated lipids. As a marker of plasma lipid peroxidation, the MDA was quantified using the method defined by Park et al. [24]. In mice treated with Triton WR-1339 (600 mg/kg), the lipid-rich plasma was removed, after that the antioxidant effect was assessed under the following conditions:

- Negative control: 40 *μ*l of plasma incubated with 40 *μ*L of distilled water;
- Positive control: 40 *μ*l of plasma produced with 10 *μ*L of a solution of CuSO_4_ (0.33 mg/mL);
- Test: 40 *μ*L of plasma incubated with 10 *μ*L of a copper sulfate solution (0.33 mg/mL) with ginger roots extracts at various concentrations;
- Standard: 40 *μ*L of plasma incubated with 10 *μ*L of a copper sulfate solution (0.33 mg/mL) with BHA at multiple levels.

After being stirred, the tubes were incubated at 50°C for 12 hours then left at ambient temperature for 60 minutes. Later, 250 *μ*L of trichloroacetic acid (TCA) (20%, pH = 3.5) and 250 *μ*L of thiobarbituric acid (TBA) (0.8%) were added to the reaction medium. After shaking, the mix was heated in a hot water bath at 95°C for one hour. After being cooled at room temperature, the tubes were mixed with 1 mL of n-butanol then shacked again and centrifuged at 4500 rpm for 15 minutes. Finally, the supernatant was collected, and the absorbance spectrophotometrically was measured at 632 nm.

### 3.5. Acute Toxicity Study

Organization for Economic Cooperation and Development guidelines 423 (acute toxic classic method, OECD guidelines for testing of chemicals, 2001) have been followed strictly for the oral critical toxicity investigation. A batch of 66 mice was divided into 11 groups, 6 mice each (3 males/3 females): the first group represents the control group, which receives the distilled water; the remaining 10 groups were treated with increasing doses of the ZOAE and the MFZO, at 2, 4, 6, 8, and 10 g/kg of body weight. After the oral administration of ZOAE and MFZO, animals were individually observed for the first 30 minutes then regularly for the early 24 hours (with a particular consideration granted for the initial 4 hours) and daily for 14 days of toxicity study.

## 4. Hypolipidemic Study of Ginger Roots Crude Aqueous Extract

### 4.1. Animals and Treatment

Adult male Albino mice weighing 25-30 g were raised in conformity with the guidelines for the Care and Use of Laboratory Animals published by the US National Institute of Health (NIH publication No. 85-23, revised 1985), in the local colonies of the Biology Department (Faculty of Sciences, Oujda, Morocco). The animals were maintained in a room, with controlled temperature (22 ± 2°C) and a photoperiod of 12 h-light: 12 h-dark. They were given food and water* ad libitum*.

### 4.2. Preparation of the High-Fat Diet

The high-fat diet was prepared with 81.8% of regular chow diet (Société SONABETAIL, Oujda, Morocco), supplemented with 2% of cholesterol and 16% of fat, 0.2% of deoxycholic acid.

### 4.3. Experimental Protocol Design

50 B6 male Albino mice were divided into five groups, each containing ten mice.

- *Group 1* represents the normolipidemic control group (NCG) and contains ten mice that receive tap water by gavage.
- *Group 2* represents the hyperlipidemic control group (HCG) and contains ten mice that receive, freely, the high-fat diet and are orally fed with tap water.
- *Group 3* represents the statin-treated group (STG) and contains ten mice that receive, freely, the high-fat diet and are orally fed with* Atorvastatin* at a dose of 10 mg/ Kg body weight/day.
- *Group 4* represents the aqueous extract treated group (AETG 1) and contains ten mice that receive, freely, the high-fat diet and are orally fed with the crude aqueous extract of* Z. officinale* at a dose of 250 mg/ Kg body weight/day.
- *Group 5* represents the aqueous extract treated group (AETG 2) and contains ten mice that receive, freely, the high-fat diet and are orally fed with the crude aqueous extract of* Z. officinale* at a dose of 500 mg/ Kg body weight/day.

It should be noted that the amount of the aqueous extract at the two doses to be administered daily orally varied according to the weight detected daily.

## 5. Biochemical Analysis

### 5.1. Plasma Analysis

The blood samples were collected using retroorbital bleeding technic, which was acceptable only as a terminal procedure while the animal was under anesthesia [25]. The fasted rats were lightly anesthetized with ether; then, the retroorbital technique was utilized to collect blood, about 1 mL were collected and put preferably into “Eppendorf” tubes that contain the citrate-citric acid as an anticoagulant (1:9* v/v*). The collected blood was centrifuged at 2000 rpm for 15 minutes, and the plasma was removed and put into another “Eppendorf” tube, which was subsequently aliquoted, numbered, subdivided into groups, and stored at -20°C until analysis.

Cholesterol and triglyceride levels in plasma were assessed by the methods reported by Allain & Kagan, [26] and Trinder [27]. Friedewald's equation was used to calculate the plasmatic LDL-C levels [28]. All biochemical tests were performed using ILab 300 chemistry analyzer (Instrumentation Laboratory Corporate Headquarters, Barcelona, Spain). The analysis was done as specified by the manufacturer's instructions. All experiments were carried out in triplicate.

### 5.2. Atherogenic Index of Plasma (AIP), LDL-C/HDL-C, and TC/HDL-C Ratios

The atherogenic index of plasma (AIP) is the logarithm of the concentration ratio of triglycerides and HDL cholesterol. Based on previous research, it is known that the AIP is associated with a higher risk of cardiovascular disease, including stroke [29]. The following formula calculated it:

(3) $$\begin{array}{c} AIP=\log\left[\;\frac{Triglycerides}{HDL-C}\;\right] \end{array}$$

The *LDL* − *C*/*HDL* − *C* and the *TC*/*HDL* − *C* ratios were computed as the ratio of plasma LDL-C to HDL-C and the plasma TC to HDL-C levels, respectively.

### 5.3. Liver Analysis

#### 5.3.1. Extraction of Hepatic Lipids

To determine the lipid content, hepatic samples were prepared from different segments of the raw liver, removed after the sacrifice of the fasted mice at the end of the experiment, after 12 weeks of treatment. A fragment of 1 gram of each mice liver was transferred into 10 mL of isopropanol solution. Maintained in an ice bath the organ was fresh ground; then, the mixture was put in a beaker and placed at 4°C for 48 hours. Later, the mixture was homogenized using magnetic stirring then centrifuged at 2500 rpm for 15 minutes. The supernatant was collected and used for the determination of the liver lipid profile. The findings were expressed as mg of cholesterol/TG per gram of body weight.

#### 5.3.2. Liver Lipid Profile

An aliquot of 10 *μ*L of the prepared supernatants was used for each test then 1 mL of total cholesterol or triglyceride reagent was added. After being homogenized and incubated at 37°C for 5 minutes, the absorbance was measured at 510 nm for cholesterol and 520 nm for TG determination. The levels of triglycerides and total cholesterol in the hepatic extracts were examined by the protocol given in the diagnostic kits previously utilized for the plasma analysis.

### 5.4. Fecal Analysis

#### 5.4.1. Extraction of Fecal Lipids

The method adopted was that of [30], which is itself based on a way reported by [31]. The fecal materials collected from fasted animals were gathered and dried at 60°C. The feces were then weighed and crushed. An amount of 1 gram of powdered feces of each mouse was extracted with 5 mL of standard saline solution and 5 mL of chloroform and methanol (2:1,* v*/*v*) and then centrifuged; the supernatant was collected, dried at 50°C, and dissolved with ethanol. Fecal TC and TG levels were analyzed with the same diagnostic kits that were used earlier for the plasma analysis.

#### 5.4.2. Fecal Lipid Levels

The fecal lipid levels were determined similarly as those of liver lipids.

### 5.5. Statistical Analysis

Our results were expressed as means ± SEM. The obtained data were analyzed by using GraphPad Prism Software, Inc. (San Diego, CA, USA) version 6.05 and using unpaired Student's t-test for statistical significance between two groups. Then, the analysis of variance (ANOVA) followed by Turkish's test was performed for the treatment studies; a “p” value less than 0.05 was considered statistically significant.

## 6. Results

### 6.1. Determination of Total Phenolic and Flavonoid Contents

The determination of total polyphenol content of* Z. officinale* crude aqueous extract and methanol extract by the Folin-Ciocalteu method showed that the crude and methanol extract contain a high concentration of polyphenols: 15.34 ± 2.21  mg/g of GAE and 27.12 ± 3.08  mg/g of GAE and for the crude aqueous extract and the methanolic fraction, respectively, as well as high flavonoids content, it was expressed as 4.20 ± 1.23  mg/g of quercetin equivalent and 11.67 ± 2.86  mg/g of quercetin equivalent for the ZOAE and the ZOMF, respectively (Table 1). It is clear from these values that the ZOMF contains more bioactive molecules than the ZOAE, either total polyphenols or flavonoids, having results entirely similar to those that Shirin Adel P. R et al. [32] found, which proved that the methanolic extract of* Z. officinale* contains more phenolic compounds than the raw aqueous extract of* Z. officinale*. Ghasemzadeh et al. [33] also found a large number of polyphenols and flavonoids in different methanolic extracts in different parts of two varieties of* Z*.* officinale*.

### 6.2. Antioxidant Activities of the Aqueous and Methanolic Extracts

#### 6.2.1. DPPH Free Radical Scavenging Activity (RSA)

Both extracts exhibited an increased inhibitory activity against the free radical DPPH, in a dose-dependent manner (data not shown). The half maximal inhibitory concentration (IC_50_) of RSA of aqueous extract and methanol fraction was 23.30 ± 1.04 *μ*g/mL and 9.78 ± 0.33 *μ*g/mL, respectively (Table 1). Even more, the RSA of the ZOMF was higher than the ZOAE.

#### 6.2.2. *β*-Carotene Bleaching Test

The *β*-carotene bleaching test of ginger extracts showed an increased inhibition in a concentration-dependent way (data not shown). The IC_50_ of aqueous extract and methanol fraction were 128 ± 9.85 *μ*g/mL and 71.55 ± 2.17 *μ*g/mL, respectively (Table 1). It should be noted that, as found in the RSA test, the inhibition rate of *β*-carotene bleaching of the ZOMF was higher than the ZOAE.

It found that the ZOMF has a lower IC_50_ than that of the ZOAE either for the DPPH test or the *β*-carotene bleaching test, a result quite logical since the ZOMF contains more bioactive molecules responsible for the observed antioxidant effects, given that there is a correlation between the content of polyphenols or flavonoids in a plant and its antioxidant effects.

#### 6.2.3. Reducing Power Assay

**Figure 1:**
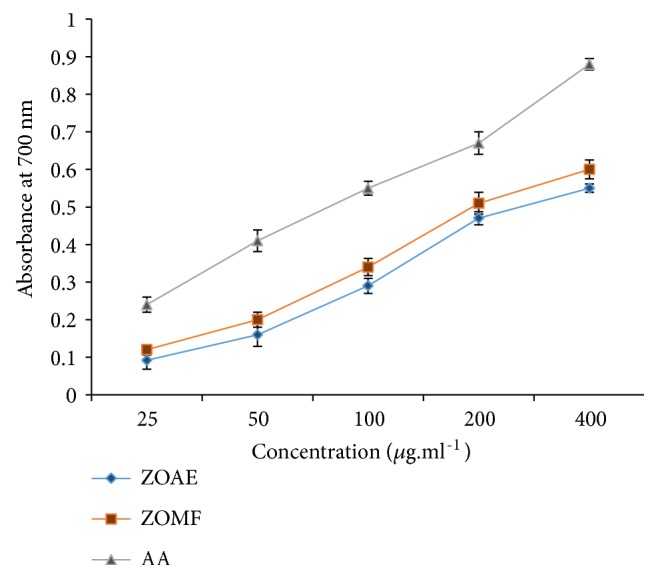
Reducing power assay of* Z. officinale* extracts. Values are mean ± SEM*; ZOAE: Z. officinale *aqueous extract; ZOMF:* Z. officinale* methanolic fraction; AA: Ascorbic acid.

The results of Figure 1 showed that the increase of the reducing powers of* Z. officinale* extracts proportionally was correlated to the augmentation of the concentration utilized. The reducing power ability of both extracts seems to be comparable to that of the positive control, the ascorbic acid.

The same observation can be noticed in the FRAP test, the ZOMF ahead of the ZOAE in antioxidant efficacy* in vitro*, because of its high content of polyphenols and flavonoids we found compared to the crude aqueous extract.

#### 6.2.4. Evaluation of Lipid Peroxidation by Assaying Thiobarbituric Acid Reactive Substances (TBARS): Dosage of Malondialdehydes

**Figure 2:**
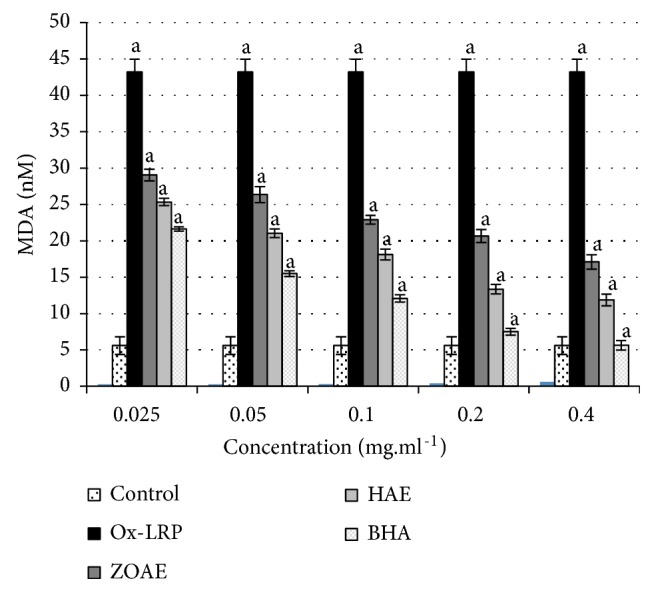
Evaluation of lipid peroxidation by assaying thiobarbituric acid reactive substances (TBARs) of* Z. officinale* extracts. Values are mean ± SEM; Ox-LRP: oxidized lipoprotein-rich plasma. ZOAE:* Z. officinale* aqueous extract; ZOMF:* Z. officinale* methanolic fraction; BHA: Butylated hydroxyanisole. a: P < 0.001; (ZOAE, ZOMF and BHA* vs.* ox-LRP and ox-LRP* vs. *control).

The evaluation of the lipid peroxidation represented in Figure 2 showed clearly that the Triton caused a significant increase in the plasma TBARS levels. It seems that the lipoprotein-rich plasma reacted with the copper sulfate when compared with the control group (+616%). Furthermore, the ZOAE and the ZOMF have a significant antioxidant effect against the CuSO_4_ action.

Figure 2 also showed that the MDA concentrations in the plasma significantly decreased after treatment with both ZOAE and ZOMF (-233.32% and -300%, respectively), at the concentration of 0.025 mg/mL, when compared to the oxidized lipoprotein-rich plasma. Both extracts showed a dose-response action on the inhibition of plasmatic levels of MDA. The lipid peroxidation profile of the BHA-treated group was expressed by a significant reduction in the plasmatic MDA.

In the TBARS test, it is noted that the BHA designated as standard showed a potent antioxidant effect, in both extracts of* Z. officinale*, both showing significant antioxidant effect, but with a better impact of the ZOMF compared to the ZOAE, having results in coherence with previous antioxidant tests (DPPH, *β*-carotene bleaching, and FRAP).

In statistical analysis, the control group was compared to the ox-LRP group, and all the ZOAE, ZOMF, and BHA groups were compared to the ox-LRP group.

### 6.3. Acute Toxicity Study

The acute oral toxicity test showed the normal behavior of the treated mice. No toxic effects were observed at a higher dose of 10 g/kg body weight. Therefore, there has been no harmful effect, and so it can be said that the ZOAE and the ZOMF are not toxic.

### 6.4. Biochemical Analysis

In all statistical studies, the NCG compared to HCG and the treated groups (AETG 1, AETG 2, and STG) were all compared to HCG.

#### 6.4.1. Weight and Daily Consumption

Table 2 shows clearly that, after the first four weeks, we observed a significant rise in mice weight when both NCG and HCG were compared. While the aqueous extract and its two doses exerted a significant inhibition of the weight increase in a dose-dependent manner, for the Atorvastatin treated group, the weight gain was lightly inhibited, and then at the 12th week, there was no significant difference and no weight gain inhibition in STG. The same results were found by Agoreyo. F. O. et al. [34], who found that the crude aqueous extract of Z. officinale exerted an inhibition of weight gain.

#### 6.4.2. Plasma Analysis

After 12 weeks of treatment, the HFD promoted a pronounced rise of plasmatic total cholesterol and triglycerides levels in comparison with the NCG (Table 3). After the first month, the TC, TG, and LDL-C levels of the HCG group were significantly increased when compared to the NCG group. Moreover, the TC, TG, LDL-C levels were further expanded up to the second month and relatively stabilized in the third month. While both doses of the aqueous extract exerted an inhibitory effect against the rise of TC, TG, and LDL-C, this effect had at once dose-dependent manner and was increasing progressively as a function of time. However, the* Atorvastatin* gave the expected impact, which is typified by the lowering of lipid parameters, principally the TC.

El-Sayed [35] found the same effects, a cholesterol-lowering effect of the crude aqueous extract of* Z. officinale* tested on Sprague-Dawley rats. In fact, by examining an infusion of* Z. officinale* at 3 doses: 100, 200, and 400 mg/kg, after 4 weeks of treatment, they found a hypolipidemic effect elucidated by a decrease in weight, TC, TG, and LDL-C, a dose-dependent and statistically significant reduction compared to Atorvastatin as the reference drug. Our results are also similar to those found by Agoreyo. F. O. et al. [34] have found a cholesterol-lowering effect of the crude aqueous extract of Z. officinale tested on Albino rats. Al-Amin et al. [36] in a study found that, at a dose of 500 mg/kg, raw ginger was significantly effective in lowering serum cholesterol levels in the ginger-treated rats.

The results of Table 4 revealed an inhibiting capability of the AIP rise. The HFD increased the AIP significantly, while this effect was reversed by treatment with ZOAE. Hence the AIP was significantly decreased, in a dose-proportional manner, upon the treatment with ZOAE, by comparison to HCG with AETG 1 and AETG 2. Likewise, the Atorvastatin tends to diminish the AIP level. Typically, the TC, TG, LDL, and the AIP improved in time.

The results presented in Table 5 demonstrated clearly that the high-fat diet significantly increased the ratios: LDL/HDL and TC/HDL of the HCG in comparison with the NCG, while the ZOAE at the doses 250 and 500 mg/kg attenuated both ratios. This antihyperlipidemic effect seems to be dose and time dependent.

Our results are in coherence with those found by S. K. Verma et al. [37], who proved that ginger extract has a significant protective effect against experimentally induced atherosclerosis in rabbits model. On their side, Jeyakumar et al. [38] also found that ginger can be concluded to have a significant hypolipidemic effect and an adverse action in the development of atherosclerosis in rats, by decreasing the increase of the concentration of lipids (tissue and serum) and lipoproteins (serum) due to an atherogenic diet.

### 6.5. Liver Analysis

**Figure 3:**
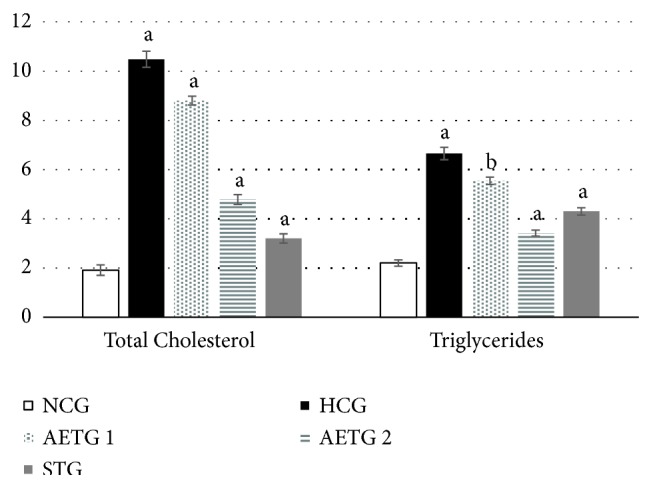
Effects of ginger extracts and Atorvastatin on total liver cholesterol and triglycerides in mice (mg/g of the liver). Values are mean ± SEM from ten mice. a: p < 0.001; b: p <0.01.

TC and TG dosage in hepatic samples represented in Figure 3 clearly showed that the HFD significantly increased both TC and TG hepatic levels by comparing the HCG to NCG. The ZOAE at both doses 250 and 500 mg/Kg significantly inhibited the increase of hepatic TC and TG, by comparing HCG to AETG 1 and AETG 2, whereas STG considerably decreased the effect of both hepatic TC and TG.

### 6.6. Fecal Analysis

**Figure 4:**
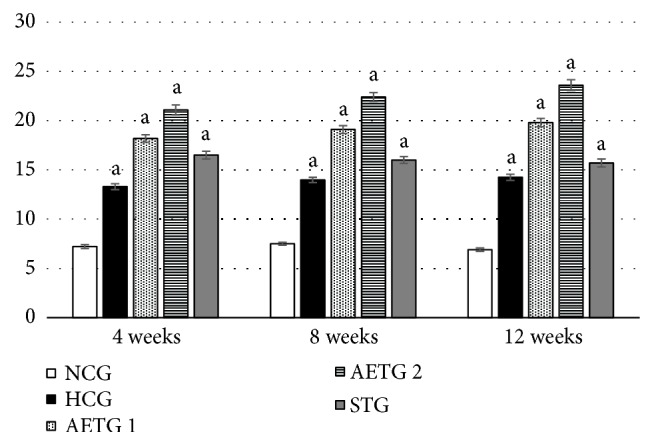
Changes on fecal total cholesterol excretion in regular and treated groups during 12 weeks (mg/g of feces).

Figure 4 shows clearly that the treatment with the ZOAE caused a remarkable enhancement of TC excretion in mice, in comparison with the NCG. Also, mice treated with a dose of 500 mg/Kg had a higher TC excretion than those administered at the dose of 250 mg/Kg. Moreover, it appears that the TC excretion in the fecal material has a dose-dependent effect. The findings also allow concluding that this effect is a time-dependent one, because of the rinsing pattern of the excretion rate throughout treatment.

## 7. Discussion

It has long recognized that a close relationship between the consumption of dietary fat and the incidence of cardiovascular diseases (CVD) is existing [39, 40]. Also, a correlation between high-fat diets and obesity is established, mainly by inducing the hypertrophy and hyperplasticity of adipocytes leading to the body weight gain [41] and subsequently the occurrence of coronary diseases [42]. Moreover, the diets overloaded with high-fats seem to be responsible for the onset of hyperlipidemia and the generation of free radicals (especially ROS and RNS), which appear to be contributing to the emergence of cardiovascular and cerebrovascular diseases besides diabetes mellitus [43, 44]. On the other hand, it has been clearly shown that the Mediterranean diet, which is highly wealthy in spices, fruits, and vegetables, is inversely proportional with the development of the cardiovascular ailments [45]. The spices and plant food such as virgin-olive, sunflower [46], celery (*A. graveolens*) [47], and garlic (*Allium sativum*) [48] were reported to prevent perfectly hyperlipidemia and atherosclerosis. Furthermore, it has been evidenced that free radicals cause oxidative changes in biomolecules such as plasma membrane constituents, DNA and LDL-C, giving rise to oxidative stress that is supposed to be the source of numerous disorders, including atherosclerosis [49].

Extensively used as a spice for over a thousand years [50, 51] and widely employed for its gustatory and facilitating qualities of digestion in both Asian and Moroccan cuisine,* Z. officinale* is also a medicinal spice with multiple properties [52]. It is also considered an essential ingredient in Ayurveda and Chinese herbal medicine for the treatment of various diseases [53].

*Z. officinale* used in the pharmaceutical industry [54, 55]. Furthermore, many studies were performed and affirmed the efficiency of* Z. officinale* to treat many affections, like nausea and vomiting [56–58]; pain and cold [59, 60]; arthritis and rheumatism [61–63]; cramps, fever, and infections [64]; gastrointestinal disorders [54, 65]; anemia [66]; and asthma, constipation, and nervous diseases [61], as well as Alzheimer's disease [64].

In the present study, we assessed the antioxidant potentials* in vivo* and* in vitro* using various assay types; we also aimed at investigating the beneficial effects of* Z. officinale* on plasma, liver, and fecal lipid profiles after chronic treatment of mice with high-fat diet over 12 weeks. In that connection, it expected that the treatment with the plant extracts would promote the recovery of normal levels of total cholesterol, low-density lipoprotein cholesterol, and triglycerides of hypercholesterolemic mice fed with fat diet. Our findings reported a high and significant* in vitro* antioxidant activity clear in all tests performed, which are all coherent with several other works, for the results of the radical scavenging test. The effects we found were the same observed by Ghasemzadeh et al., [33] who found a significant scavenging activity of the methanolic extracts in different parts of two varieties of* Z*.* officinale* compared to BHT. Also, Khalaf et al. [67] have found an important radical scavenging activity of* Z. officinale *methanolic extract compared to the AA. Our results were also comparable to those found by Shirin Adel P. R & Prakash [32] who found that both aqueous and methanolic extracts of* Z. officinale* have an important radical scavenging activity, with better efficiency of the methanolic extract than that of the aqueous extract. For the *β*-carotene bleaching test, the antioxidant effect observed by Soher E. Ali et al. [68] is perfectly consistent with ours; in fact, Soher E. Ali et al. [68] found that ginger extracts possessed antioxidant activities via the *β*-carotene/linoleate system. Concerning the FRAP test, the results we obtained were consistent with those found by Shirin Adel P. R & Prakash, [32]; indeed, they found that the crude aqueous extract and the methanolic extract both had significant antioxidant activity by FRAP method, with a greater effect in the methanolic extract than in the crude aqueous extract, results perfectly consistent with those we found. Regarding the TBARs test results, similar findings to ours were discovered in several other works such as that of Stoilova et al. [69] and Munasinghe et al. [70], who all proved that the ginger extracts showed an antioxidant activity comparable with that of BHT in inhibiting the lipid peroxidation at a high temperature. In addition, Si et al. [71], Ghasemzadeh et al. [33], Nile & Park [53], and also Maizura M. et al. [72] all found high antioxidant activities in* Z. officinale* extracts, but the most important point is that they have arrived at establishing a correlation between polyphenols contents and the antioxidant actions. Likewise, the study noticed a significant hypolipidemic effect substantiated by a notable decline in plasma lipid profile (LDL-C, TG, and TC) of the ZOAE (250 mg/Kg and 500 mg/kg); identical findings were discovered in several other works such as [8, 73, 74] and also [75] who all have reached a common conclusion, which is that the* Z. officinale* have a very noticeable hypolipidemic effect on high-fat-fed mice and rats. Similar effects were observed in liver and feces lipid profiles; the achieved results, especially those of the plasmatic lipid profile, have allowed us to bring out some other parameters: AIP, HDL/LDL, and TC/LDL ratios, which are sensitive parameters related to cardiovascular risks, including atherosclerosis, ischemia, and stroke. The obtained results have shown that the* Z. officinale* extracts exert a significant inhibitory effect on the AIP, HDL/LDL, and TC/LDL ratios increasing. All these effects were dose and time dependent, for three months of follow-up. The observed beneficial effects in the current report may be attributed to the high content of polyphenols and flavonoids contained in the aqueous and methanol extracts of* Z. officinale*. These findings seem to be in compliance with those found by [33, 53, 58] and also those found by [54], who all have approved the high content of polyphenols in* Z. officinale* extracts. And more precisely, gingerols, shogaols, paradols, and zingerone were confirmed by [71]; moreover, Nile & Park [53] approved that ginger extracts were very rich in phenolic compounds, and mainly 6-gingerol, 6-shogaol, and 6-paradol; Li et al. [54] also have shown that extracts of* Z. officinale* contain a variety of bioactive molecules, mainly zingerone, 6-gingerol, 8-gingerol, 10-gingerol, and 6-shogaol, which are certainly responsible for the antioxidant, hypolipidemic, and antiatherosclerotic effects. Chrubasik et al. [76] affirmed on their side that the raw ginger contains up to 9% lipids or glycolipids and about 5–8% oleoresin. The pungent principle, accounting for 25% of the oleoresin, consists mainly of gingerols. 6-Gingerol (the main gingerol) is more pungent than 8-gingerol or 10-gingerol. Other gingerols include methylgingerol and gingerdiol, dehydrogingerdione, 10-dehydrogingerdione, gingerdiones, diarylheptanoids (equivalent to curcuminoids, e.g., hexahydrocurcumin), diterpene lactones, and galanolactone (in some species).

These results are also highly consistent with reports of the Sabatini [77], showing that the aqueous and methanolic extracts have cholesterol-restrictive capabilities and capacity to mitigate the hastened atherosclerosis in hypercholesterolemic development subjects. In our study, we found that the flavonoids and the polyphenols are present in both the aqueous and the methanolic extracts of the* Z. officinale*, which can most likely be responsible for their hypolipidemic and antiatherogenic effects. Also, the ginger crude is constituted by bioactive molecules such as 6-gingerol [78], zingerone [79], phenolic 1,3-diketones [80], and the 6-paradol [81], which have been shown to protect against lipid peroxidation in various established models. Moreover, He & Huang [82] identified polyphenols and antioxidants by HPLC and chromatographic methods coupled with mass spectrometry. They also evidenced a series of pharmacological activities, including the hypocholesterolemia and antioxidant. Another study showed the ability of polyphenols isolated from green tea to inhibit the LDL oxidation by macrophages in culture. Also, catechin and quercetin inhibited the oxidation of LDL-C when they were tested with different cells in cultures, such as human monocyte-derived macrophages, endothelial cells of a human umbilical vein, or lymphoid cells [83]. It has been suggested that this phenomenon was because of the capability of flavonoids to block the lipoxygenase activity* in vivo* and* in vitro* [84, 85].

Additionally, to obstruct cell-mediated LDL-C oxidation, quercetin and catechin likewise managed to block the cytotoxic effects of the oxidized LDL on the lymphoid cells, likely due to the increase of antioxidants present in these flavonoids [86]. Total phenols content correlated with the antioxidant power of many plant extracts [87]. Nonetheless, our research did not find a significant enhancement of HDL levels by* Z. officinale* extracts in the HFD-mice, yet the LDL-C/HDL-C and TC/HDL-C ratios significantly reduced in a dose-dependent way. This result can be exploited taking into account the interest of the LDL-C/HDL ratio as an indicator of cardiovascular diseases, knowing that this parameter is closely associated with the hazard of cardiovascular disorders, even when TC rates raised [88] and that the TC/HDL-C ratio represents a responsive indicator of atherosclerosis [89].

In this respect, the small LDL-C/HDL-C and TC/HDL-C ratios observed in the mice fed with high-fat diet and treated with* Z. officinale* extract propose that these extracts have potentially an antiatherogenic action, precisely like [75] who proved that the ginger extract possessed hypolipidemic, antioxidant, and anti-inflammatory properties and therefore is very promising for the treatment of CVD in humans. These results suggest that* Z. officinale* extracts have a substantial therapeutic effect for better management of hyperlipidemia, through the avoidance of the atherogenic, coronary, and cardiovascular disorders. Additionally, these data may be justified by the raising of LDL-C catabolism by provoking the transfer of cholesterol derived from all tissues to the liver, intended for secretion in the form of bile acids. Our findings reveal that the bioactive compounds incorporated in this plant have a polar nature because of their water solubility. This discovery was harmonious with preceding reports proving that polar plant extracts had cholesterol-restrictive capabilities and the capacity to decrease the increased atherosclerogenesis in hypercholesterolemic animal models [90].

These results can be judged conclusive for the hyperlipidemia-induced atherosclerosis treatment and seemingly prove the traditional utilization of ginger roots for hyperlipidemic cases all over the world generally, and especially in Morocco. However, further studies are necessary to elucidate the exact mechanisms of the* Z. officinale* effect on plasma, liver, and fecal lipid parameters.

## 8. Conclusion

In conclusion, our results show that the aqueous extract and the methanolic fraction of* Z. officinale* exert remarkable antioxidant activities as well as a potent hypolipidemic and antiatherogenic effects in mice without potential acute toxicity. However, further studies should be done to confirm our findings, and so the exploitation of* Z. officinale* extracts in favor of the valorization process, in hope to use it as a diet supplement for hypercholesterolemic people.

## Figures and Tables

**Table 1:** Polyphenols and flavonoid contents and antioxidant activities (RSA and *β*-carotene) of *Z. officinale.*

	*Z. officinale* extracts		Standards	
	ZOAE	ZOMF	AA	BHA
Polyphenols (mg eq gallic acid/g Ext)	15.34 ± 2.21	27.12 ± 3.08	-	-
Flavonoids (mg eq quercetin/g Ext)	4.20 ± 1.23	11.67 ± 2.86	-	-
RSA (IC_50_ (*μ*g/mL))	23.30 ± 1.04^b^	9.78 ± 0.33^b^	1.83 ± 0.01^a^	-
*β*-carotene (IC_50_ (*μ*g/mL))	128.41 ± 9.85^b^	71.55 ± 2.17^b^	-	2.20 ± 0.05^a^

All values were expressed as mean ± standard error of the mean; a: expressed as mg gallic acid equivalent/g of dry plant extract; b: expressed as mg quercetin equivalent/g of dry plant extract. a: p < 0.001; c: p < 0.05; b: p < 0.01; NS: not significant.

**Table 2:** Comparison of weight gain and food intake from the 4th week to the 12th week.

	*4th week *		*8th week*		*12th week*	
	Weight (g)	Food intake (g/mouse/day)	Weight	Food intake (g/mouse/day)	Weight	Food intake (g/mouse/day)
NCG	25.2 ± 0.4	3.15 ± 0.08	25.8 ± 0.2	2.95 ± 0.10	26.1 ± 0.2	3.01 ± 0.12
HCG	32.5^a^ ± 0.2	2.93 ± 0.11	33.3^a^ ± 0.3	3.11 ± 0.06	34.8^a^ ± 0.3	3.14 ± 0.08
AETG 1	27.4^b^ ± 0.2	3.02 ± 0.05	29.9^c^ ± 0.2	2.99 ± 0.05	30.6^c^ ± 0.2	2.92 ± 0.09
AETG 2	27.0^b^ ± 0.3	3.10 ± 0.09	28.5^b^ ± 0.1	2.85 ± 0.10	29.1^c^ ± 0.2	3.04 ± 0.11
STG	28.6^c^ ± 0.4	2.98 ± 0.04	29.7^c^ ± 0.2	3.07 ± 0.08	31.5^NS^ ± 0.4	2.89 ± 0.03

Values are expressed as means ± SEM from ten mice in each lot. a: p < 0.001; c: p < 0.05; b: p < 0.01; NS: not significant.

**Table 3:** Changes of plasma, total cholesterol, and triglycerides (mmol.L^−1^) in control and treated mice.

	4th week		8th week		12th week	
	TC	TG	TC	TG	TC	TG
NCG	2.10 ± 0.11	1.07 ± 0.06	2.35 ± 0.15	1.21 ± 0.05	2.51 ± 0.13	1.48 ± 0.08
HCG	4.62 ± 0,25^a^	2.49 ± 0.13^a^	4.78 ± 0.22^a^	2.52 ± 0.12^a^	4.97 ± 0.30^a^	2.83 ± 0.10^a^
AETG dose 1	3.29 ± 0.19^a^	2.25 ± 0.07^NS^	3.77 ± 0.18^b^	2.33 ± 0.1^NS^	3.80 ± 0.12^b^	2.45 ± 0.11^c^
AETG dose 2	2.36 ± 0.09^a^	1.61 ± 0.04^a^	2.42 ± 0.11^a^	1.75 ± 0.07^a^	2.55 ± 0.10^a^	1.92 ± 0.08^a^
STG	2.20 ± 0.05^a^	1.93 ± 0.05^a^	2.35 ± 0.08^a^	2.03 ± 0.07^b^	2.41 ± 0.11^a^	2.20 ± 0.13^b^

Values are expressed as means ± SEM from ten mice in each lot; TC: total cholesterol; TG: triglycerides. HCG compared with NCG. AETG and STG compared with HCG. a: p < 0.001; c: p < 0.05; b: p < 0.01; NS: not significant.

**Table 4:** Changes of plasma HDL-C, LDL-C (mmol.l^−1^), and atherogenic index of plasma in control and treated mice.

	4th week			8th week			12th week		
	HDL	LDL	AIP	HDL	LDL	AIP	HDL	LDL	AIP
NCG	1.41 ± 0.06	0.48 ± 0.08	0.49	1.45 ± 0.05	0.66 ± 0.07	0.62	1.43 ± 0.13	0.78 ± 0.04	0.76
HCG	1.02 ± 0,04^a^	2.90 ± 0.13^a^	2.79	0.88 ± 0.03^a^	3.20 ± 0.11^a^	3.43	0.91 ± 0.02^a^	3.49 ± 0.14^a^	4.46
AETG dose 1	1.36 ± 0.05^a^	1.49 ± 0.05^a^	1.42	1.43 ± 0.08^a^	1.87 ± 0.04^a^	1.64	1.45 ± 0.05^a^	1.86 ± 0.10^a^	1.62
AETG dose 2	1.42 ± 0.04^a^	0.62 ± 0.06^a^	0.66	1.49 ± 0.02^a^	0.58 ± 0.09^a^	0.62	1.55 ± 0.04^a^	0.62 ± 0.08^a^	0.65
STG	1.29 ± 0.03^a^	0.53 ± 0.09^a^	0.71	1.35 ± 0.06^a^	0.59 ± 0.08^a^	0.74	1.41± 0.06^a^	0.56 ± 0.09^a^	0.71

Values are expressed as means ± SEM from ten mice in each lot. TC: total cholesterol; TG: triglycerides; AI: atherogenic index. HCG compared with NCG. AETG and STG compared with HCG. a: p < 0.001; c: p < 0.05; b: p < 0.01; NS: not significant.

**Table 5:** Comparison of LDL/HDL and TC/HDL ratios followed for twelve weeks.

	4th week		8th week		12th week	
Ratio	LDL/HDL	TC/HDL	LDL/HDL	TC/HDL	LDL/HDL	TC/HDL
NCG	0.34	1.49	0.45	1.62	0.54	1.75
HCG	2.37	3.79	2.96	4.43	3.83	5.46
AETG1	1.08	2.42	1.30	2.63	1.28	2.62
AETG2	0.43	1.66	0.38	1.62	0.40	1.64
STG	0.41	1.70	0.43	1.74	0.39	1.71

## Data Availability

No data were used to support this study.

## Abbreviations

AA: Ascorbic Acid
AETG 1: Aqueous Extract Treated Group dose 1 (250 mg/Kg)
AETG 2: Aqueous Extract Treated Group dose 2 (500 mg/Kg)
AETG: Aqueous Extract Treated Group
AIP: Atherogenic Index of Plasma
BHA: Butylated Hydroxy Anisole
CVD: Cardiovascular Disease
DPPH: 1,1-Diphenyl Picryl Hydrazyl
GAE: Gallic Acid Equivalent
HCG: Hyperlipidemic Control Group
HDL: High-Density Lipoprotein
HFD: High-Fat Diet
LDL: Low-Density Lipoprotein
MDA: Malondialdehyde
NCG: Normal Control Group
STG: Statin-Treated Group
TBARs: Thiobarbituric Acid Reactive species
TC: Total Cholesterol
TG: Triglycerides
RSA: Radical Scavenging Activity
ZOAE: * Zingiber Officinale* Aqueous Extract
ZOMF: * Zingiber Officinale* Methanolic Fraction.

## Additional Points

*Practical Applications.* Our results can be very beneficial for hyperlipidemic people to treat hyperlipidemia and all cardiovascular complications since the results showed that the extracts of* Z. officinale* rhizome possess both antioxidant and hypolipidemic effect, two effects accompanied, becoming closely related to the phenomenon of atherosclerosis. Also, the hypolipidemic effect observed was dose and time dependent, and this was at the plasmatic, hepatic, and fecal levels. Finally, we can say that our study provided a scientific basis for the prevention of hyperlipidemia through a dietary approach.
